# CircRNAs in Xiang pig ovaries among diestrus and estrus stages

**DOI:** 10.1186/s40813-022-00270-1

**Published:** 2022-06-23

**Authors:** Xi Niu, Yali Huang, Huan Lu, Sheng Li, Shihui Huang, Xueqin Ran, Jiafu Wang

**Affiliations:** Institute of Agro-Bioengineering / Key Laboratory of Plant Resource Conservative and Germplasm Innovation in Mountainous Region and Key Laboratory of Animal Genetics, Breeding and Reproduction in the Plateau Mountainous Region (Ministry of Education), College of Life Science and College of Animal Science, Guizhou University, Guiyang, 550055 China

**Keywords:** Xiang pig, Ovary, Estrus, CircRNA, Expression profile, CeRNA network

## Abstract

**Background:**

The fecundity of sows is a trait of major economic in pig industry. The molecular regulation of estrus cycles can affect the fecundity of female animals. Compared with the other pig breeds, Xiang pig exhibits the special estrus behaviors. CircRNAs are thought to involve in regulation of multiple biological processes. However, the potential roles of circRNAs in ovary regulation on Xiang pig estrus are largely unknown.

**Results:**

8,937 circRNAs were identified from eight libraries constructed from the ovarian samples of Xiang pig at estrus and diestrus stages by RNA sequencing method. Of which, 1,995 were high confidence circRNAs detected at least two junction reads in each ovary sample and seven circRNAs were validated by RT-PCR method. Furthermore, we identified 290 upregulated and 15 downregulated circRNAs in estrus ovaries. These differentially expressed circRNAs (DECs) derived from 273 host genes. And 207 miRNAs were identified to be targets sponged by 156 DECs with 432 binding sites, containing more than one miRNA binding site in each circRNA. Function enrichment analysis revealed that the host genes and the targets of miRNAs sponged by DECs were enriched in several reproduction-related signaling pathways, such as ovarian steroidogenesis, oocyte maturation, circadian rhythm, estrogen signaling pathway, GnRH signaling pathway, circadian entrainment, and oocyte meiosis. The circRNA-miRNA-mRNA networks revealed that 153 miRNAs interacting with 122 DECs and 86 miRNAs interacting with 84 DECs were involved in ovarian functions and ovarian circadian entrainment and circadian rhythm respectively. The DEC-miRNA-DEG (differentially expressed gene, DEG) networks associated with reproduction-related signaling pathways contained 22 DECs,18 miRNAs and 7 DEGs. 22 DECs were recognized as hub circRNAs during the estrus phase of Xiang pigs.

**Conclusions:**

The circRNAs that function as miRNA sponges could play a key role in post-transcriptional regulation of gene expression during Xiang pig’s estrus cycle.

**Supplementary Information:**

The online version contains supplementary material available at 10.1186/s40813-022-00270-1.

## Background

Pork is the main meat consumed in human. Indigenous pigs contribute important part of pork production. Sow fecundity is pivotal in pig industry to ensure pork production amount, which is much diverse between pig breeds [1]. Ovary function directly influences the fecundity of female animals [2]. During each estrus cycle, the ovary undergoes a series of complex biological processes in morphological, hormonal, and biochemical changes. These biological processes in ovary are involved in the transcriptional and post-transcriptional regulation of many genes [3]. Previous studies have revealed that non-coding RNAs, including a variety of lncRNAs, miRNAs, and circRNAs are widely involved in post-transcriptional regulation of gene expression and various biological processes [4].

CircRNAs are a type of non-coding RNA with a covalently closed loop structure, which are derived from the back-spliced exonic or intronic sequences [5]. CircRNAs are abundant in the cytoplasm and can be co-expressed with the linear transcripts from which they are derived [6]. A great number of circRNAs have been identified in a variety of eukaryotic organisms by RNA-seq method [5]. CircRNAs contain miRNA binding sites and may function as miRNA sponges, or as transcriptional activators. Furthermore, circRNAs have been shown to segregate RNA binding proteins [7], and can even become translated into proteins through cap-independent translation initiation. Additionally, many evidences suggest that circRNAs are involved in a wide range of biological processes and function as ceRNA, which can influence the expression level of their parental genes [8, 9]. Recently, many studies using deep-sequencing approaches have reported that circRNAs are differentially expressed in ovary between pig breeds [8, 9]. Breed-specific circRNAs could be potentially associated to reproduction traits [10, 11].

**Fig. 1:**
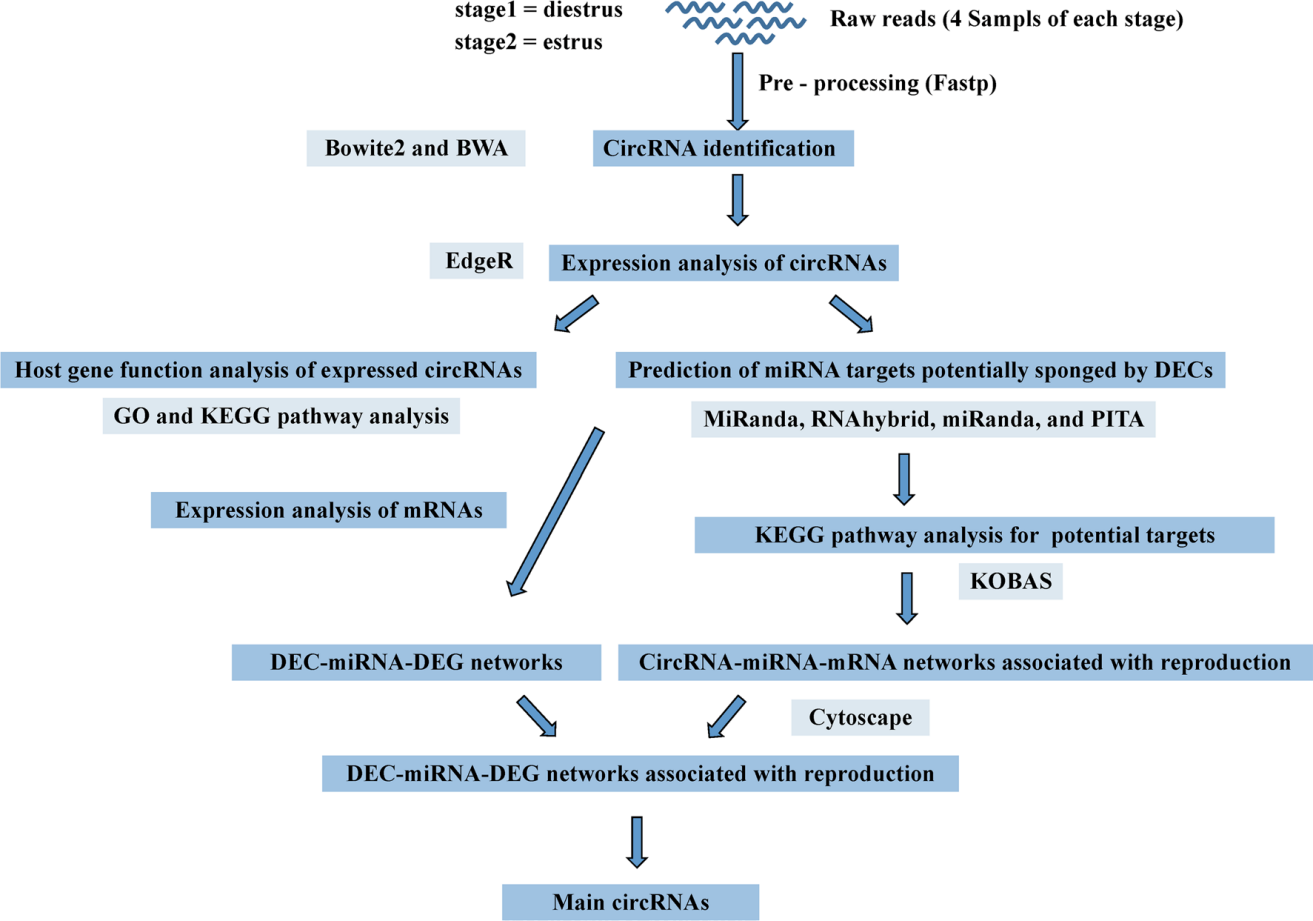
The flow chart of present research

Xiang pig is a miniature indigenous pig breed originated from the southeast in Guizhou province of China, and the meat were the dominant dietary intake sources for the mountain resident populations. It is featured by small size, early sexual maturity, lower litter size, excellent meat quality, and not clear exhibition of estrus behaviors [2]. The exhibition of estrus behaviors in Xiang gilts or sows was not very clearer than in Meishan or other European pigs [10, 11]. The difference on estrus expression between pig breeds could be affected by genes and could be improved by selection [12]. However, the potential roles of circRNAs in ovary regulation on Xiang pig estrus are largely unknown. The investigation of circRNAs in Xiang pig ovary may provide a valuable opportunity to understand the molecular basis of pig reproduction. To obtain more knowledge on the roles of circRNAs in Xiang pig ovary during estrus cycle, we performed a genome-wide analysis of transcripts of ovary tissues from Xiang pig sows at diestrus and estrus phases using RNA sequencing method. We investigated the expression profiles of circRNAs in ovarian tissues and identified differentially expressed circRNAs (DECs) (Fig. 1). Our results suggest that circRNAs probably participate in the regulation of gene expression in pig’s estrus cycle and reproduction.

## Results

### Characterization of circRNAs expressed in Xiang pig ovaries at estrus and diestrus

**Fig. 2:**
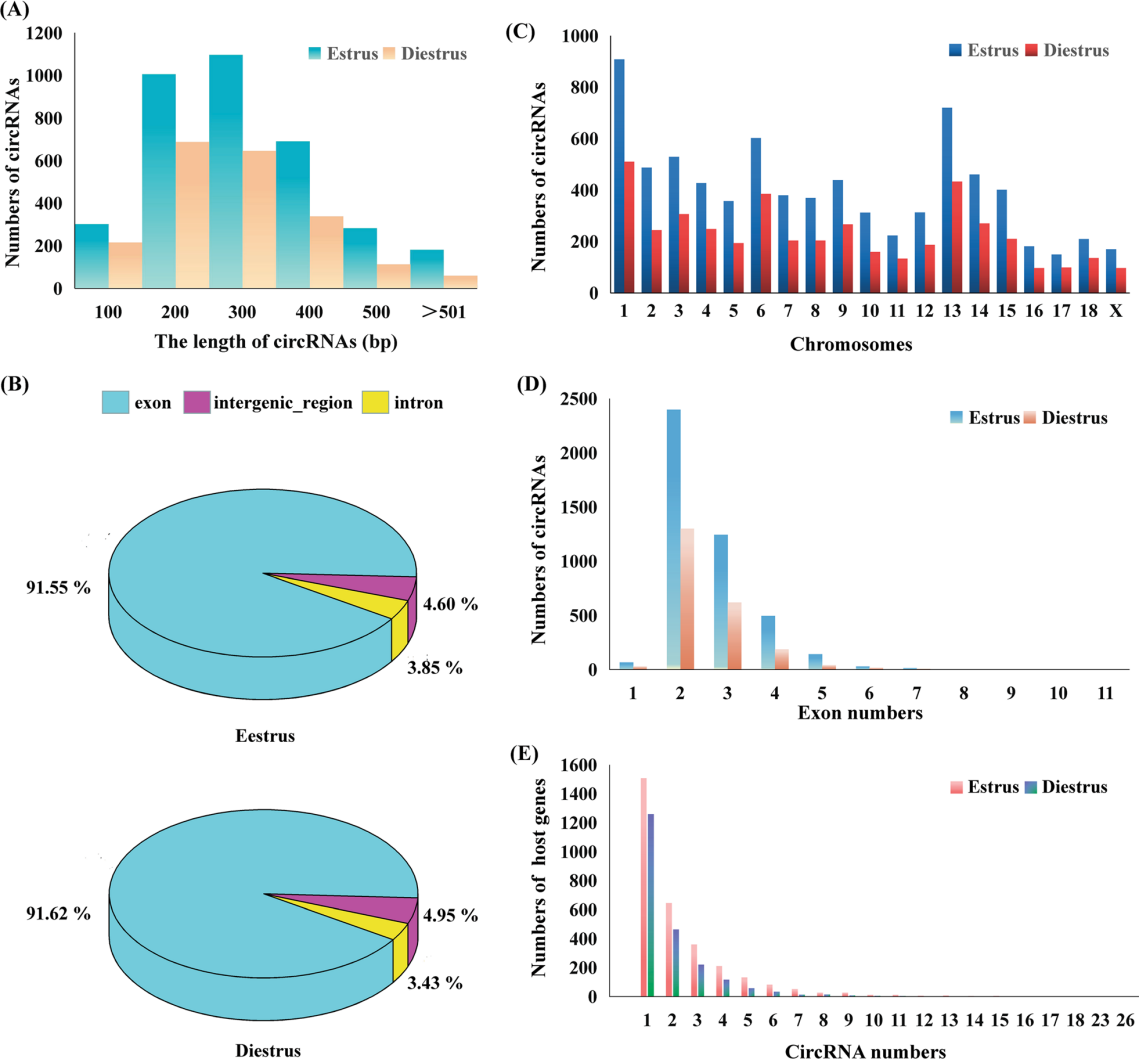
Characterization of circRNAs expressed in Xiang pig ovaries at estrus and diestrus. **A** Amounts of circRNAs with different length. **B** Distribution of circRNAs in genic regions. **C** Distribution of circRNAs in chromosomes. **D** Exon numbers contained in circRNAs. **E** Characteristics of host genes producing circRNAs

To uncover the expression and function of circRNAs in porcine ovary, we performed total RNA sequencing in adult ovaries of Xiang pig during estrus and diestrus. The sequencing of the RNA libraries generated about 152 million 100-bp paired-end reads and an average of 124.3 million mapped reads per sample (Additional file 1: Table S1). We used find_circ and CIRI2 for circRNA detection and identified circRNAs with a junction reads greater than 2, which were detected in at least one sample of the biological replicates. We detected 4,404 circRNAs from the diestrus group, 7,680 circRNAs from the estrus group. In total, 8,937 unique circRNAs were detected from both software in two group samples (Additional file 2: Table S2), whereas 3,147 circRNAs were co-expressed in ovaries between two stages. The length of these circRNAs were shorter than 2 kb, which varied from 32 to 1,814 bases in estrus group, 29 to 1,146 bases in diestrus group, but mainly enriched between 100–400 bases (Fig. 2A). The composition and sources of these circRNAs between estrus and diestrus samples did not present significant difference. The percentages of the circRNAs that derived from exons, introns, and intergenic regions in estrus samples were 91.55%, 4.21%, and 4.6%, respectively, while they were 91.62%, 3.43%, and 5.4% in diestrus samples, respectively (Fig. 2B). According to their parental gene locations, these circRNAs were widely distributed on 1–18 autosomes, X chromosome, and mitochondria (Fig. 2C). It was found that the numbers of circRNAs from Chromosome 1, 6, and 13 were more than that from other chromosomes. Most (> 98%) circRNAs were derived from multiple exons, of which circRNAs with 2–3 exons accounted for > 87.3% within the same parental gene (Fig. 2D). Very few circRNAs were composed of more than 5 exons. The most exon composition (n = 11) was found in circ_2424 (host gene, *ASPH*). Additionally, 42.59% and 51.2% of the parental genes generated more than one circRNA in ovary samples at estrus and diestrus, respectively (Fig. 2E). We found that the gene DNA helicase (*CHD*2) had 28 predicted circRNAs.

### Analysis of DECs in Xiang pig ovaries between estrus and diestrus

**Fig. 3:**
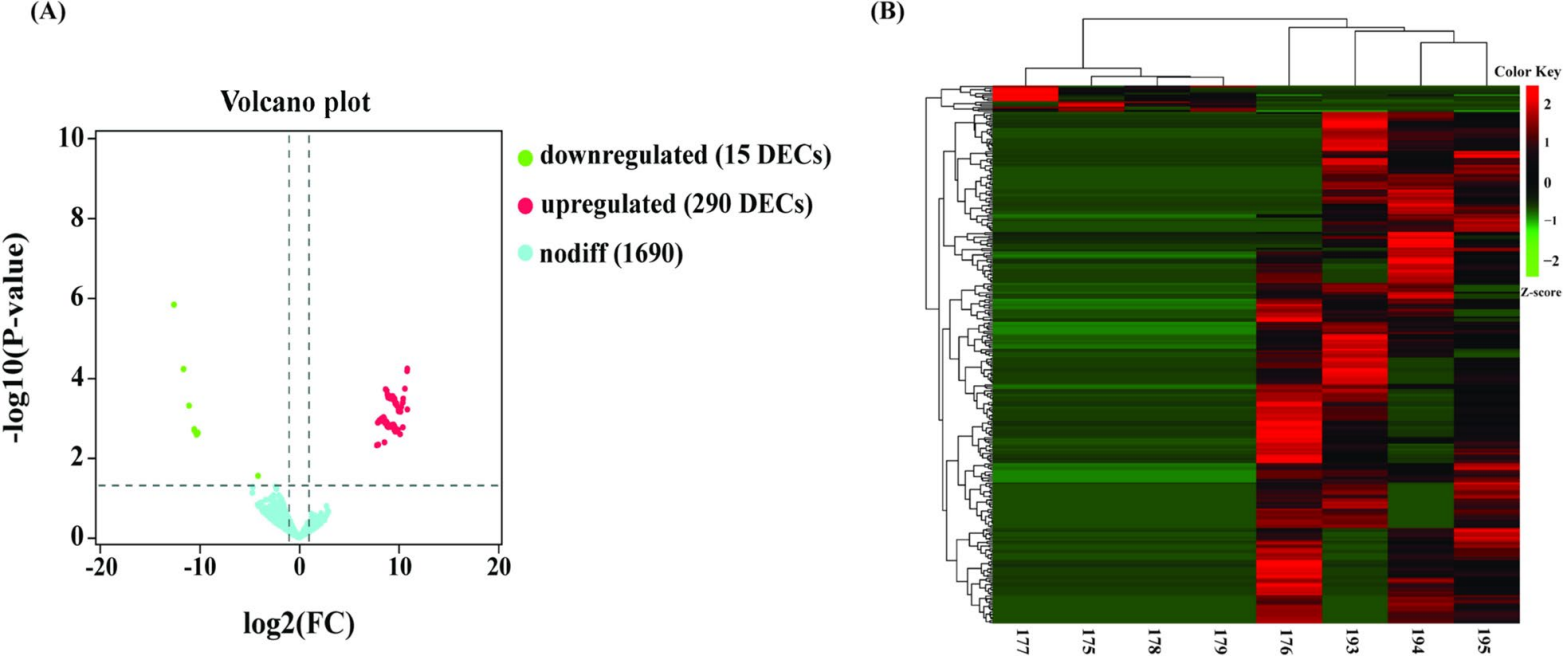
Volcano plot and heatmap analysis of DECs. **A** Volcano plot of DECs. **B** Heatmap of DECs Diestrus samples: 175,177,178, and 179; Estrus samples:193, 194,195, and 176

To evaluate the dynamic changes of circRNA expression in Xiang pig ovaries between estrus and diestrus, we focused on circRNAs that were detected at least two junction reads in each biological replicate of one specific tissue for high confidence. This analysis yielded 1,995 high confidence circRNAs. We performed differential expression analysis on the high confident circRNAs with edgeR. Compared with diestrus ovaries, 305 DECs were detected from estrus ovaries. Of these, 294 DECs were derived from 273 host genes (*P* < 0.05) with 290 upregulated and 15 downregulated DECs (Fig. 3A, Additional file 3: Table S3). In addition, we clustered the DECs in both diestrus and estrus ovaries. As shown in the heatmap (Fig. 3B), samples at the same stages were clustered together, and the expression levels of circRNAs exhibited dynamic changed during estrus cycle.

### CircRNAs validation by RT-PCR method

**Fig. 4:**
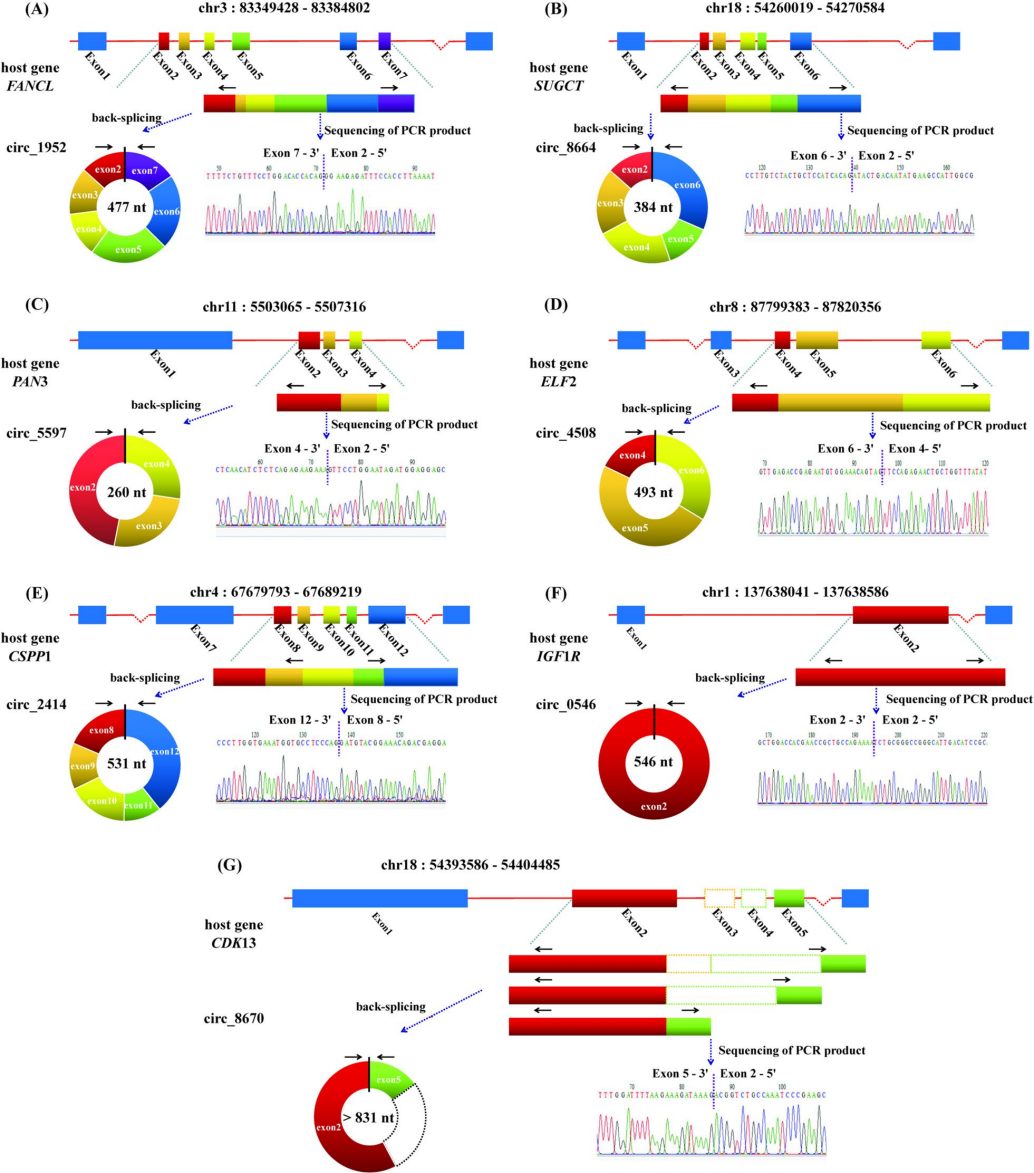
Diagram of circRNAs generation by a way of back-splicing and the sequence detected by Sanger method. **A** Circ_1952 was generated from exon 2, 3, 4, 5, 6, and 7 of gene *FANCL*. **B** Circ_8664 was generated from exon 2, 3, 4, 5, and 6 of gene *SUGCT*. **C** Circ_5597 was produced from exon 2, 3, and 4 of gene *PAN*3. **D** Circ_4508 was produced from exon 4, 5, and 6 of gene *ELF*2. **E** Circ_2414 was derived from exon 8, 9, 10, 11, and 12 of gene *CSPP*1. **F** Circ_0546 was derived from exon 2 of gene *IGF*1*R*. **G** Circ_8670 was generated at least from exon 2 and 5 of gene *CDK*13

To validate the reliability of predicted circRNAs, seven circRNAs were randomly chosen from the top 50 of CPM value in 1,995 high confidence circRNAs at estrus and diestrus phases and verified the region of spliced junction by RT-PCR method using a pair of divergent primers. These circRNAs consisted of 6 exons (circ_1952), 5 exons (circ_8664, circ_2414), 3 exons (circ_5597, circ_4508), 2 exons (circ_8670) and 1 exon (circ_0546) respectively. In Fig. 4A–G, the circularized states and the determined sequence at the junction of the 7 circRNAs were presented. The junction regions of circRNAs sequenced by Sanger method were consistent with those putative analysis of circRNAs by RNA-seq.

### Host gene function analysis of expressed circRNAs

**Fig. 5:**
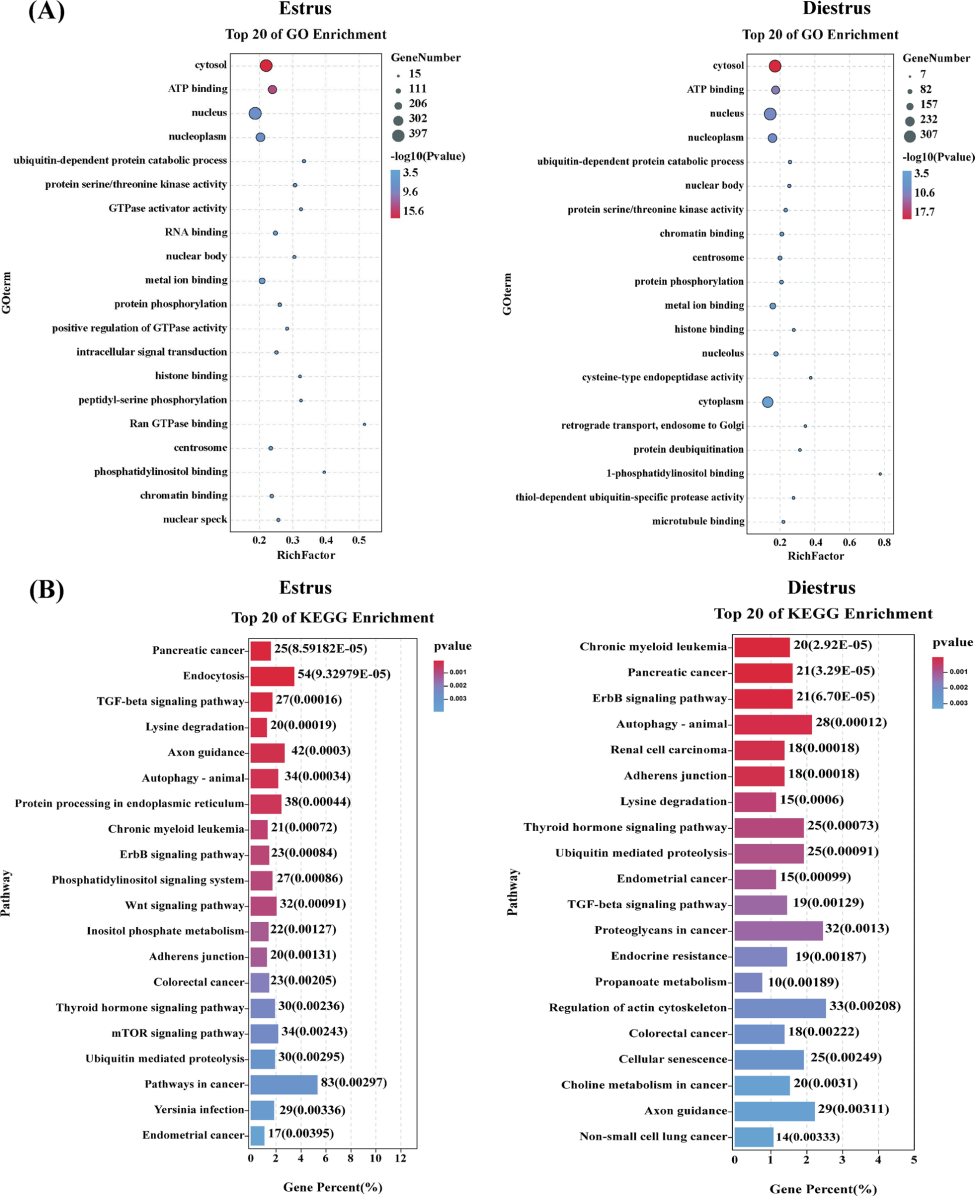
Go and KEGG analysis for host genes of circRNAs at estrus and diestrus phases. **A** Go functional analysis. **B** KEGG enrichment analysis

To understand the biological functions of circRNAs, we performed GO and KEGG pathway analysis to predict the functions of circRNAs in Xiang pig ovaries during diestrus and estrus (Additional file 4: Table S4). GO analysis indicated that the host genes of circRNAs were significantly associated with cellular metabolic process, cellular component organization or biogenesis, regulation of catabolic process, biological regulation, binding and activity, and regulation of transcription by RNA polymerase II (*P* < 0.05) (Fig. 5A). Pathway analysis indicated that the host genes of circRNAs were involved in valine, leucine and isoleucine degradation, propanoate metabolism, human T-cell leukemia virus 1 infection, endocytosis, progesterone-mediated oocyte maturation, protein processing in endoplasmic reticulum, Fc epsilon RI signaling pathway, mitophagy–animal, and neurotrophin signaling pathway (*P* < 0.05) (Fig. 5B).

### Prediction of miRNA targets potentially sponged by DECs

In this study, we used miRanda v3.3 and RNAhybrid v1.2 to predict the targeted miRNAs of DECs. We identified 432 binding sites in 156 DECs to bind with 207 miRNA molecules by all two prediction programs (Additional file 5: Table S5, Additional file 16: Fig. S1). We found that any one of circRNAs could contain 1–5 miRNA-binding sites. Accordingly, the same miRNA may bound with multiple circRNAs.

**Fig. 6:**
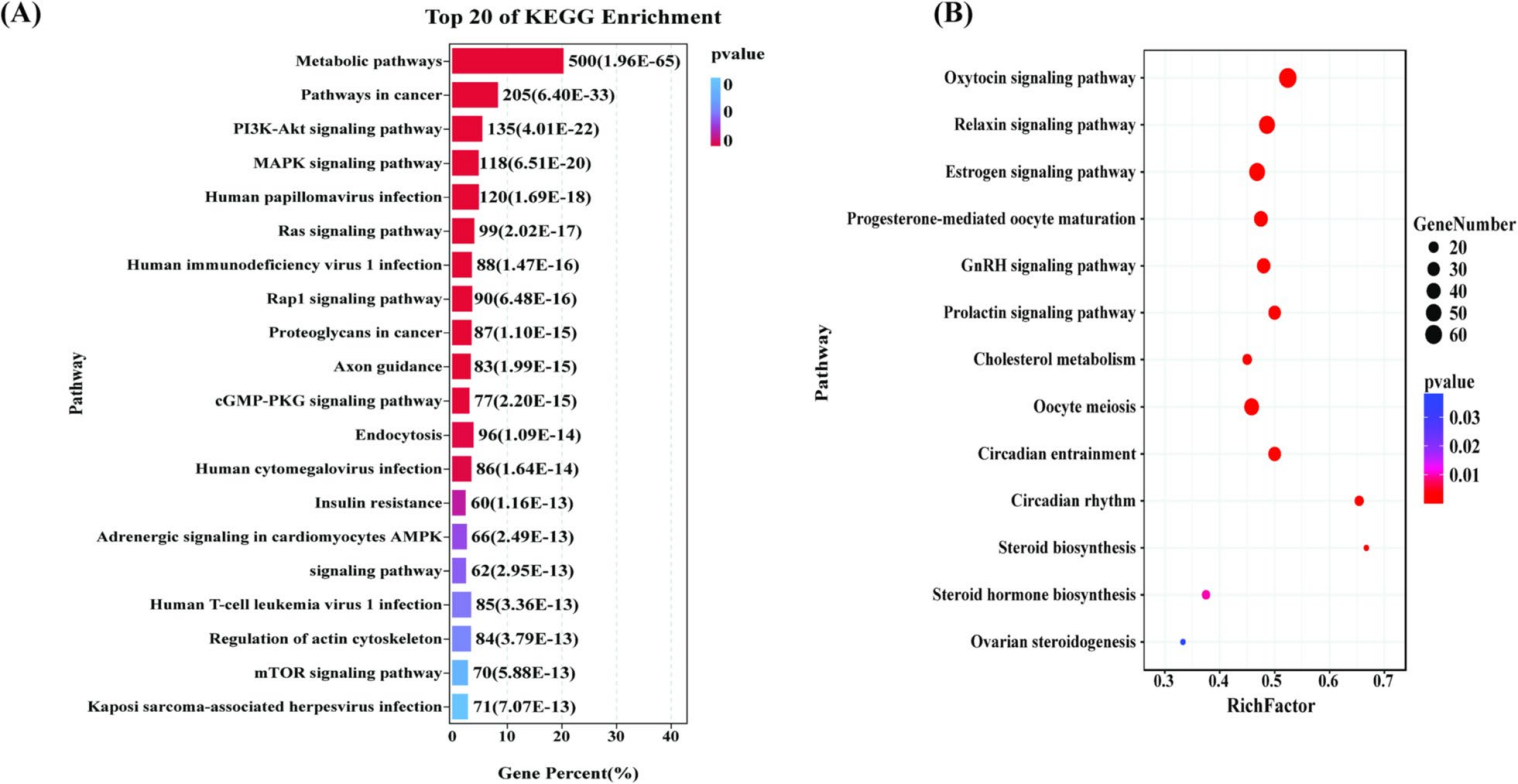
KEGG enrichment analysis for protein-coding target genes of miRNAs sponged by DECs. **A** The top 20 of KEGG enrichment. **B** The KEGG pathways were related ovarian functions

Many reports have proposed a ceRNA hypothesis that circRNAs can act as endogenous “miRNA sponge”, thereby modulating the expression of miRNA targets [7–9]. To acquire more knowledge concerning the biological role of DECs in ovary function, regulatory targets of those miRNAs sponged by DECs were predicted by miRanda v3.3 and PITA in the present study. A total of 9,307 potential targets (Additional file 6: Table S6) for the 207 miRNAs that bound by 156 DECs were predicted. Pathway analysis showed these predicted targets were involved in 292 possible KEGG pathways (*P* < 0.05) (Additional file 7: Table S7), including diseases (n = 85), signal transduction (n = 50), metabolism (n = 34), cell cycles, apoptosis, cell communication, and other pathways. The top 20 of KEGG enrichment were showed in Fig. 6A. Many pathways were associated with ovarian functions (Fig. 6B). Significantly, several pathways were associated with reproduction, such as oocyte meiosis, circadian entrainment, circadian rhythm, ovarian steroidogenesis, estrogen signaling pathway, progesterone-mediated oocyte maturation, and GnRH signaling pathway. Therefore, we further analyzed the interaction between circRNAs, miRNAs, and mRNAs of these pathways associated with reproduction in detail (Additional file 8: Table S8, Additional files 17, 18, 19, 20, 21, 22, 23: Figs. S2–S8). The circRNAs-miRNAs-mRNAs interactive networks involved in these KEGG pathways were constructed based on ceRNA mechanism. The resulting circRNA-miRNA-mRNA interactive networks provided nodes and linkages between circRNAs and their targets. Comparison of the circRNA-miRNA-mRNA networks between diestrus and estrus groups revealed that 153 miRNAs interacting with 122 DECs associated with ovarian functions and 86 miRNAs interacting with 84 DECs related with ovarian circadian entrainment and circadian rhythm (Additional file 9: Table S9).

**Fig. 7:**
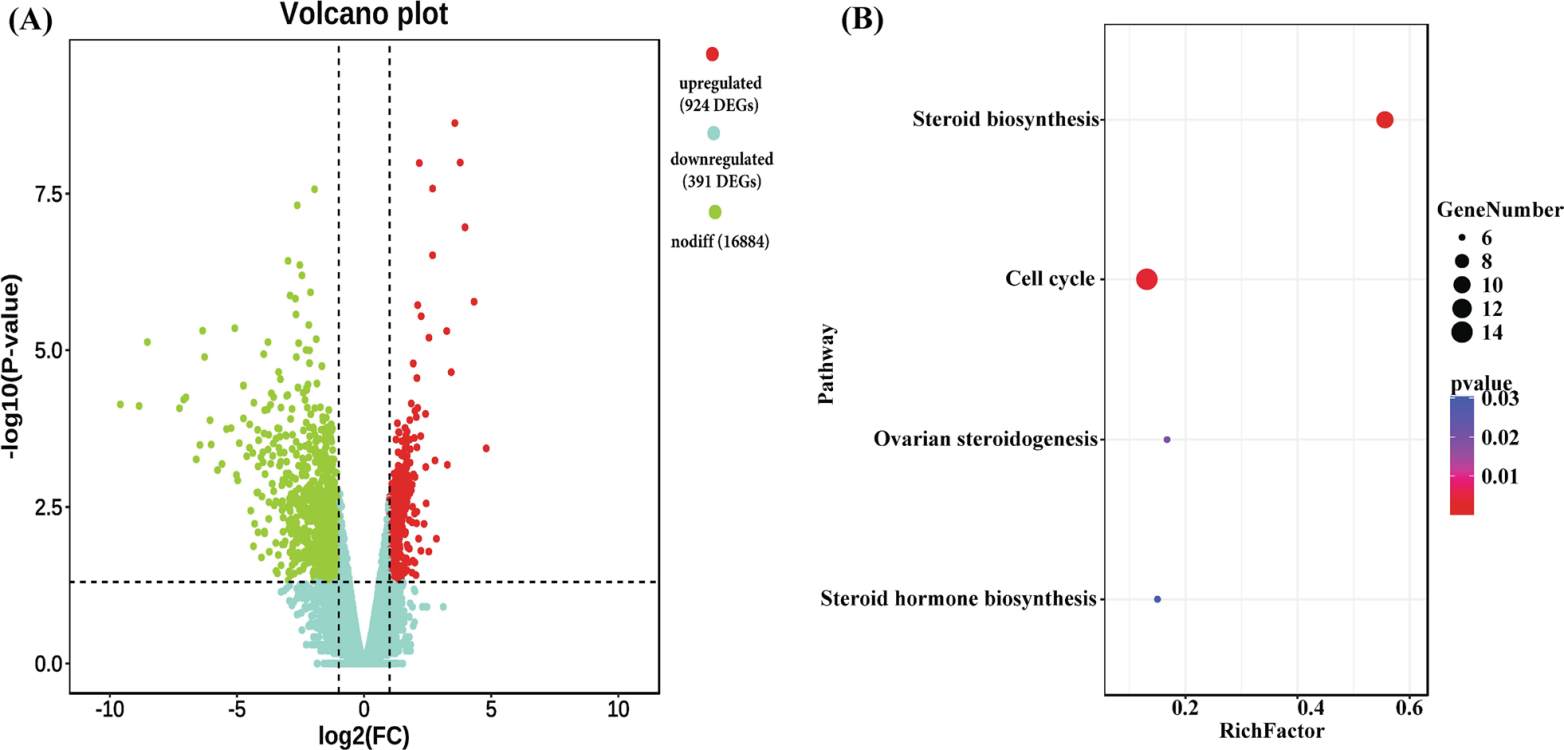
Volcano plot and the KEGG pathways of ovarian functions for DEGs. **A** Volcano plot of DEGs. **B** The KEGG pathways of ovarian functions for DEGs

**Fig. 8:**
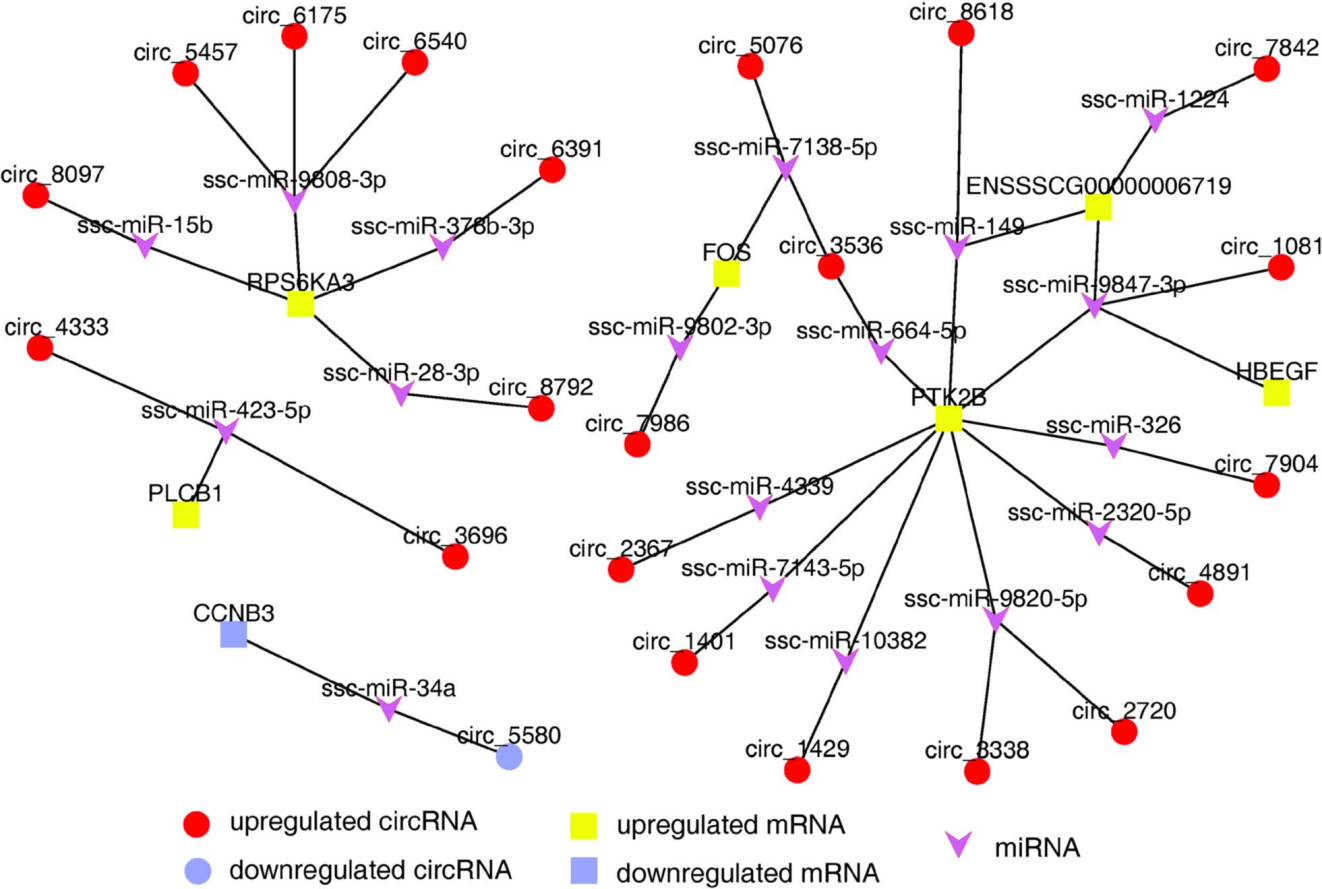
The DEC-miRNA-DEG networks associated with reproduction pathways

To further understand the role of the key circRNAs in the regulation of gene expression during estrus stage, we performed the analysis of DEGs and constructed the DEC-miRNA-DEG interactive networks. At gene level, we identifieda total of 1,315 genes differentially expressed in ovaries between estrus and diestrus phases (Additional file 10: Table S10, Fig. 7A), of which 924 genes were upregulated and 391 genes were downregulated in estrus ovaries. Pathway analysis using KOBAS program showed DEGs participated in 51 possible KEGG pathways (*P* < 0.05) (Additional file 11: Table S11). Significantly, four pathways were associated with reproduction (Fig. 7B), such as steroid biosynthesis, cell cycle, ovarian steroidogenesis, and steroid hormone biosynthesis. And then we had predicted the 137 DECs-182 miRNAs-571 DEGs interactive networks (Additional file 12: Table S12). We found that 175 miRNAs which targeted 531 upregulated genes were sponged by 130 upregulated circRNAs and 12 miRNAs that also targeted 40 downregulated genes were competed by 7 downregulated circRNAs. At the same time, we further analyzed the interaction between DECs, miRNAs, and DEGs of above-mentioned pathways associated with reproduction (oocyte meiosis, circadian entrainment, circadian rhythm, ovarian steroidogenesis, estrogen signaling pathway, progesterone-mediated oocyte maturation, and GnRH signaling pathway). The DEC-miRNA-DEG networks associated with reproduction contained 22 DECs, 18 miRNAs, and 7 DEGs (Fig. 8, Additional file 13: Table S13).

## Discussion

Ovary is an important reproductive organ of female animal. They provide fertile oocytes, secrete reproductive hormones, and maintain the estrus cycle of female animals. In each estrus cycle, the ovary undergoes changes of proliferation, invasion, differentiation, and apoptosis, which involve in the transcriptional regulation of many genes. These physiological changes directly affect or determine the ovulation, fertilization rate and litter size of animals [13]. CircRNAs are a new class of endogenous non-coding RNAs that have been found to be widely expressed in human and animal cells and function in many biological processes [5, 14, 15]. Previous studies indicate that circRNAs are involved in regulation and may serve as novel regulators of ovarian follicle growth and development during porcine reproduction processes [10, 16–18]. In this study, we used RNA-seq to investigate the circRNA profiles in ovaries of Xiang pig sows during diestrus and estrus phases. We identified 8,937 circRNAs in the ovaries from diestrus and estrus groups. The circRNAs generated from estrus ovaries were more than that from diestrus ovaries. However, there were 3,147 circRNAs were co-expressed in ovaries between two stages. We found that over 91% of circRNAs were comprised of exonic sequences, containing one or more exons. Furthermore, about 5% of the circRNAs were generated from intergenic regions. The length of these circRNAs mainly enriched between 100 and 400 bases. These observations were similar to previous findings in pig ovaries [19]. It suggested that the circRNAs might play a key role in ovarian functions for Xiang pig.

Furthermore, 42.59 ~ 51.2% of the parental genes generated more than one circRNA in ovary samples at estrus and diestrus. The most predicted circRNAs (n = 28) were derived from the gene *CHD*2 (Table S14). Genetic studies demonstrate that Chromatin remodeling enzymes play critical roles in organizing genomic DNA within the native chromatin state. *CHD*2 is a member of the chromodomain helicase DNA-binding family of proteins and regulates gene expression through chromatin remodeling. Chd2-deficient mice have been demonstrated to exhibit a general growth delay and perinatal lethality [20]. These studies suggest that *CHD*2 plays an intrinsic role in normal mammalian development [21, 22]. In our study, we obtained 9 high confidence circRNAs from 28 predicted circRNAs derived from *CHD*2, of which 3 circRNAs were differentially expressed between estrus and diestrus ovaries. However, we didn’t predict any putative miRNA binding sites for these 3 DECs. These indicated these DECs derived from *CHD*2 do not act as miRNA sponges. Their function in ovaries should be further studied.

We identified 305 DECs (Additional file 3: Table S3). The identification of these DECs demonstrated that there was a larger difference in expression and regulation of genes between diestrus and estrus ovaries. At gene level, a total of 1,315 genes differentially expressed in ovaries between estrus and diestrus phases (Additional file 10: Table S10). By comparison with the expression in diestrus ovaries, 924 genes were upregulated and 391 genes were downregulated in estrus ovaries. Of the 273 host genes for 305 DECs, we found that only 20 host genes were differentially expressed. These observations suggested that gene expression during estrus cycle was regulated at different levels and the expression of circRNAs was highly regulated and controlled.

It is generally accepted that the functions of circRNAs are related to the functions of their parent genes [6]. Previous studies have indicated that many differentially expressed coding and noncoding RNAs are widely expressed in the diestrus or estrus stages and several genes could affect estrus of animals [23]. In our study, we found that eighteen genes, which have known to be involved in ovary functions or other reproductive processes, produced more than one circRNAs (Additional file 3: Table S3). For instance, *CPEB*1, *MAPK9*, *PIK*3*CA*, *KDM*1*A*, *Dicer*1, and *FBXL*3 contained two to three predicted circRNAs, respectively. These genes participate in several signaling pathways to regulate steroid hormone biosynthesis, ovarian circadian rhythm, oocyte development and maturation [24]. Several circRNAs (circ_3954, circ_1268, circ_6675, circ_6675, circ_4235, and circ_5758) were significantly upregulated in estrus ovary compared with diestrus ovary. The results illustrated that the circRNAs produced by these genes might involve in hormone biosynthesis, oocyte maturation and estrus cycle maintenance.

Even now the function of circRNAs is not well understood [25]. Many studies have revealed that some circRNAs can serve as a miRNA sponge and subsequently suppress its activity to regulate gene expression, or cross-talk with transcriptional machinery [7, 26].

We predicted 432 miRNA targeted by 156 of 305 DECs (Additional file 5: Table S5). Each circRNA contains one or more miRNA binding sites. For example, circ_1968 sponged ssc-let-7a, ssc-let-7d-5p, and ssc-let-7f-5p. Circ_2456 sponged ssc-miR-21-3p. Circ_2141 contained 9 potential binding sites to 4 different miRNAs, including ssc-miR-34a, ssc-miR-133a-5p, ssc-miR-138, and ssc-miR-7137-3p. Circ_1268 harbored two binding sites with ssc-miR-191. Circ_7558 functioned as sponges for ssc-miR-132 and ssc-miR-374b-5p. Previous studies have indicated that numerous miRNAs involved in regulating female reproductive hormone signaling during estrus [27–30]. MiR-191, miR-132, miR-370, and miR-181a were found to be associated with follicular development. Furthermore, miR-19b, miR-21, miR-31, miR-106a, and miR-224 were associated with follicular granule cell development. MiR-133 has been demonstrated to regulate oocyte meiosis and suppress ovarian cancer cell proliferation [31, 32].

To fully understand the biological role of miRNAs sponged by DECs in ovary function, we analyzed the targets of the miRNAs sponged by DECs. Pathway analysis showed that these predicted targets participate in a lot of KEGG pathways, including several pathways associated with reproduction, such as oocyte meiosis, circadian entrainment, circadian rhythm, ovarian steroidogenesis, estrogen signaling pathway, progesterone-mediated oocyte maturation, GnRH signaling pathway. Compare of the circRNA-miRNA-mRNA networks between diestrus and estrus groups revealed that 153 miRNAs interacting with 122 DECs associated with ovarian functions, and 86 miRNAs interacting with 84 DECs related with ovarian circadian entrainment and circadian rhythm processes (Additional file 9: Table S9).

To further illustrate the role of the circRNAs in the regulation of gene expression during estrus stage, we constructed the DEC-miRNA-DEG interactive networks (Additional file 12: Table S12). These networks revealed that so much circRNAs were interacted with miRNAs and may act as ceRNAs to medicate the expression of miRNA targets. Many of the target genes, such as *PTK*2*B*, *RPS*6*KA*3, *CCNB*3, *PLCB*1, *FOS,* and *HBEGF*, were associated with reproduction pathways. Thus, we hypothesized that these cirRNAs, miRNAs, and mRNAs in the DEC-miRNA-DEG networks could play critical roles in estrus regulation. For example, we found that thirteen circRNAs (circ_1429, circ_7842, circ_8618, circ_4891, circ_7904, circ_2367, circ_3536, circ_5076, circ_1401, circ_7986, circ_2720, circ_3338, and circ_1081) were interacted with twelve miRNAs (ssc-miR-10382, ssc-miR-1224, ssc-miR-149, ssc-miR-2320-5p, ssc-miR-326, ssc-miR-4339, ssc-miR-664-5p, ssc-miR-7138-5p, ssc-miR-7143-5p, ssc-miR-9802-3p, ssc-miR-9820-5p, and ssc-miR-9847-3p) as ceRNAs to medicate the expression of the related genes such as *HBEGF*, *PTK*2*B*, *FOS,* and ENSSSCG00000006719. Previous reports have shown that *HBEGF* may actively control the process of follicle growth and maturation in the zebrafish [33]. *PTK*2*B* can encode a cytoplasmic protein tyrosine kinase which was reported to participate in the development of the final stages of follicles and ovary development in Hu sheep [34]. And *FOS*, a critical downstream mediator of PGR and EGF signaling, plays an important role in ovulation [35]. In our study, the expression of *HBEGF*, *PTK*2*B,* and *FOS* gene were upregulated in estrus ovaries and the expression patterns were consistent with the thirteen circRNAs motioned above. The results showed that the ovary needs to express enough proteins, such as HBEGF, PTK2B, and FOS to meet the biological processes of follicular development and oocyte maturation during estrus cycle. Therefore, in order to remove the translation inhibition of target mRNA by the miRNAs, the cells would increase the expression level of related circRNAs as a miRNA sponge, thereby inhibiting the post transcriptional regulation of target mRNA by miRNAs.

Although some circRNAs function as miRNA sponges, 149 of 305 (48.85%) Xiang pig circRNAs identified in this study have no putative miRNA binding site. Several studies have suggested that most circRNAs do not act as miRNA sponges and they have functions including regulation of host gene transcription, protein binding, and translation [7–9].

## Conclusions

In conclusion, our results demonstrated that ovaries generated abundant circRNAs during estrus, of which numerous circRNAs were differentially expressed between diestrus and estrus phases. We predicted 432 miRNA targets by 156 DECs. Each circRNA can contain one or more miRNA binding sites. Function enrichment analysis revealed that their host genes and the targets of miRNAs sponged by DECs were enriched in several reproduction-related signaling pathways, such as ovarian steroidogenesis, oocyte maturation, circadian rhythm, estrogen signaling pathway, GnRH signaling pathway. The DEC-miRNA-DEG networks associated with reproduction-related signaling pathways contained notes of 22 DECs, 18 miRNAs, and 7 DEGs. 22 DECs were recognized as hub circRNAs during the estrus phase of Xiang pigs. These results suggest that circRNAs probably participate in the regulation of gene expression in pig’s estrus cycle and reproduction.

## Materials and methods

### Sample preparation

Xiang pig sows after weaning were obtained from the Guizhou Dachang pig breeding company, Guizhou, China. The animal preparation and estrus detection were referred to the methods of Tang et al. (2018) and Ran et al. (2021) [2, 36]. Four animals from each group were regarded as biological replicates. The animals from post-weaning sows were monitored twice daily for behavioral estrus. On Day 10 and on Day 20 after estrus, the sows were considered in the diestrus and estrus phase, respectively. The ovarian samples were collected with surgery at 10 days before expected estrus and the day of the third estrus when the sows exhibited strong performance of reddening and swelling of the vulva. All samples were immediately frozen in liquid nitrogen and stored at − 80℃ until RNA extraction.

### Library construction and RNA sequencing

The total RNA samples were isolated from ovarian tissues at diestrus and estrus stages using TRIzol reagent (Invitrogen, Carlsbad, CA, USA) according to the manufacturer's instructions. The quantity and integrity of the total RNAs were analyzed using a NanoDrop 2000 (Thermo Fisher Scientific Inc., Waltham, MA, USA) and an Agilent 2100 Size Bio-analyzer system (Agilent Technologies, CA, USA). The values of RNA integrity number (RIN) > 7.0 were used for RNA-seq analysis. About 5 μg total RNAs per sample were used for sequencing library preparation using a NEBNext® Ultra™ Directional RNA Library Prep Kit for Illumina® (NEB, Ipswich, MA, USA). The ribosomal RNAs were removed from the total RNA using a Ribo-Zero™ GoldKits (Epicentre, Madison, WI, USA). The remaining RNA was fragmented and reverse-transcribed according to the description of TruSeq RNA LT/HT sample preparation kit (Illumina, USA). After the quality of the cDNA libraries were qualified by Bioanalyzer 2200 evaluation (Agilent, Santa Clara, CA), sequencing was conducted on an Illumina HiSeq 2500 instrument (Illumina, San Diego, CA, USA).

### CircRNA identification

Firstly, the reads were removed that contained low-quality, adaptor and more than 5% unknown nucleotides via Fastp software [37]. The remaining high quality reads were then used for subsequent analysis. These reads were aligned to the reference genome of Sus scrofa (Sscrofa11.1) by employing Bowite2 or BWA [38, 39]. Bam files of unmapped reads from Bowite2 and sam files of mapped reads from BWA were input to find_circ and CIRI2, respectively. The candidates from two softwares were intersected to obtain the final circRNAs dataset based on chromosome location. The sequence splicing of circRNAs and the visualization of ring construction diagram were performed by using CIRI-full and CIRI-vis, respectively [40].

### Expression analysis of circRNAs and mRNAs

The different expression patterns of circRNAs and mRNAs were calculated by edgeR package [41]. The reads numbers mapped to each circRNA were counted and the average of two softwares find_circ and CIRI2 was taken as expression level of each circRNA transcript. It was worth noting that the junction reads in a circRNA were greater than or equal to 2 in each sample. All CPM values of circRNA were added 0.1 for logarithm arithmetic. CPM = (circRNA read counts * 10^6^) / the sum of circRNAs read counts [14]. The protein-coding gene expression level was counted by featureCounts software [42]. The expression level of mRNA was estimated by CPM value. CPM = (Count of reads mapped to a mRNA * 10^6^) / Total count of mapped reads from the library [43]. The thresholds of DECs and DEGs were |log2 (fold_change)|≥ 1 and *P* < 0.05 [16].

### Prediction of miRNA targets and circRNA-miRNA-mRNA network construction

Target miRNAs of DECs were predicted via MiRanda v3.3 (http://www.microrna.org/microrna/home.do) and RNAhybrid v1.2 (http://bibiserv.techfak.uni-bielefeld.de/rnahybrid). The intersections results from miRanda and RNAhybrid was identified as the target miRNAs with the minimal free energy of − 10 kcal/mol. MiRNAs sequences of pig were originated from miRBase database (http://www.mirbase.org/). The target mRNA by miRNA were determined as the shared mRNA between results from miRanda and PITA (http://genie.weizmann.ac.il/pubs/mir07/mir07_dat-a.html) with a set of minimal free energy to be − 10 kcal/mol. The diagram of circRNA-miRNA-mRNA regulatory interaction networks was depicted via Cytoscape software [44].

### Go and KEGG analysis

To analyze function of circRNAs, Go and KEGG analysis were performed by KOBAS online [45], in which host genes of circRNAs and target genes of miRNAs sponged by DECs were taken as input. The established criteria (*P* < 0.05) was considered to indicate significant enrichment.

### Validation of circRNAs by Sanger sequencing

Most of identified circRNAs were expressed at low levels with CPM value less than 1,000 especially those DECs between the estrus and diestrus phases. To confirm the reliability of the predicted circRNAs from RNA sequencing, seven circRNAs were randomly chosen from those circRNAs with CPM value (average CPM > 2500) ranked at the top 50. These seven circRNAs included circ_1952, circ_8664, circ_5597, circ_4508, circ_2414, circ_0546, and circ_8670. The information (eg: CPM, FDR, and so on) of those circRNAs was showed in Additional file 15: Table S15 in manuscript. The difference of these 7 circRNAs levels was not significant between estrus and diestrus stages. Moreover, we confirmed the reliability of the predicted circRNAs from RNA sequencing by verifying the region of spliced junction through RT-PCR method using a pair of divergent primers flanking the BSJ. The region of BSJ of circRNA is composed of a canonical 5′ splice site sequence joined to an upstream 3′ splice site sequence [46]. The PCR products were analyzed by 2% agarose gel electrophoresis and further proofed via Sanger sequencing (Sango Biotech, Shanghai, China).

## Supplementary Information

**Additional file 1. Table S1**. Overview of RNA-Seq data.

**Additional file 2. Table S2**. The circRNAs expressed in the ovaries of Xiang pig at diestrus and estrus phases.

**Additional file 3. Table S3**. High confident circRNAs and DECs between diestrus and estrus ovaries.

**Additional file 4. Table S4**. GO and KEGG for host genes of circRNAs in estrus and diestrus.

**Additional file 5. Table S5**. The target miRNAs sponged by DECs.

**Additional file 6. Table S6**. Target genes of miRNAs sponged by DECs.

**Additional file 7. Table S7**. GO and KEGG for target genes of miRNAs sponged by DECs.

**Additional file 8. Table S8**. Interaction of circRNA-miRNA-mRNA.

**Additional file 9. Table S9**. CircRNAs and miRNAs in pathways involves in ovarian function.

**Additional file 10. Table S10**. The DEGs between diestrus and estrus ovaries.

**Additional file 11. Table S11**. GO and KEGG for DEGs.

**Additional file 12. Table S12**. Interaction of DEC-miRNA-DEG.

**Additional file 13. Table S13**. Interaction of DEC-miRNA-DEG associated with reproduction pathways.

**Additional file 14. Table S14**. CircRNAs were derived from the gene *CHD*2.

**Additional file 15. Table S15**. The information of verified 7 circRNAs.

**Additional file 16. Fig. S1.** Prediction of miRNA targets potentially sponged by DECs.

**Additional file 17. Fig. S2.** The circRNA-miRNA-mRNA interactive networks of circadian rhythm pathway.

**Additional file 18. Fig. S3.** The circRNA-miRNA-mRNA interactive networks of circadian entrainment pathway.

**Additional file 19. Fig. S4.** The circRNA-miRNA-mRNA interactive networks of estrogen signaling pathway.

**Additional file 20. Fig. S5.** The circRNA-miRNA-mRNA interactive networks of GnRH signaling pathway.

**Additional file 21. Fig. S6.** The circRNA-miRNA-mRNA interactive networks of progesterone-mediated oocyte maturation pathway.

**Additional file 22. Fig. S7.** The circRNA-miRNA-mRNA interactive networks of oocyte meiosis pathway.

**Additional file 23. Fig. S8.** The circRNA-miRNA-mRNA interactive networks of ovarian steroidogenesis pathway.

## Data Availability

The data supporting the conclusions of this article are included within the article and its addition files. The raw sequencing data are available from SRA database in NCBI with accession number PRJNA835060.

## Abbreviations

CircRNA: Circular RNA
DEC: Differentially expressed circRNA
MiRNA: MicroRNA
MRNA: Messenger RNA
ceRNA: Competitive endogenous RNA
LncRNA: Long noncoding RNA
*ASPH*: Aspartate-β-hydroxylase
*CHD*2: Chromodomain helicase DNA binding protein 2
GO: Gene ontology
KEGG: Kyoto encyclopedia of genes and genomes
GnRH: Gonadotropin-releasing hormone
*FANCL*: FA complementation group L
*SUGCT*: Succinyl-CoA:glutarate-CoA transferase
*PAN3*: Poly(A) specific ribonuclease subunit PAN3
*ELF*2: E74 like ETS transcription factor 2
*CSPP*1: Centrosome and spindle pole associated protein 1
*IGF*1*R*: Insulin like growth factor 1 receptor
*CDK*13: Cyclin dependent kinase 13
*CPEB*1: Cytoplasmic polyadenylation element binding protein 1
*MAPK9*: Mitogen-activated protein kinase 9
*PIK*3*CA*: Phosphatidylinositol-4,5-bisphosphate 3-kinase catalytic subunit alpha
*KDM*1*A*: Lysine-specific histone demethylase 1A
*Dicer*1: Dicer 1, ribonuclease III
*FBXL*3: F-box and leucine rich repeat protein 3
DEG: Differentially expressed gene
RT-PCR: Reverse transcription-polymerase chain reaction
BSJ: Back-spliced junction
*HBEGF*: Heparin-binding epidermal growth factor-like growth factor
*PTK*2*B*: Protein tyrosine kinase 2 beta
*RPS*6*KA*3: Ribosomal Protein S6 Kinase A3
*CCNB*3: Cyclin B3
*PLCB*1: Phospholipase C Beta 1
*FOS*: Fos proto-oncogene, AP-1 transcription factor subunit
*PGR*: Progesterone receptor
*EGF*: Epidermal growth factor
PLC: Phospholipase C
RNA-seq: RNA sequencing
