# Supplementary figures and images for: CircRNAs in Xiang pig ovaries among diestrus and estrus stages
Source: Porcine Health Manag. 2022 Jun 23;8:29. doi: 10.1186/s40813-022-00270-1 (PMC9219244; doi:10.1186/s40813-022-00270-1)

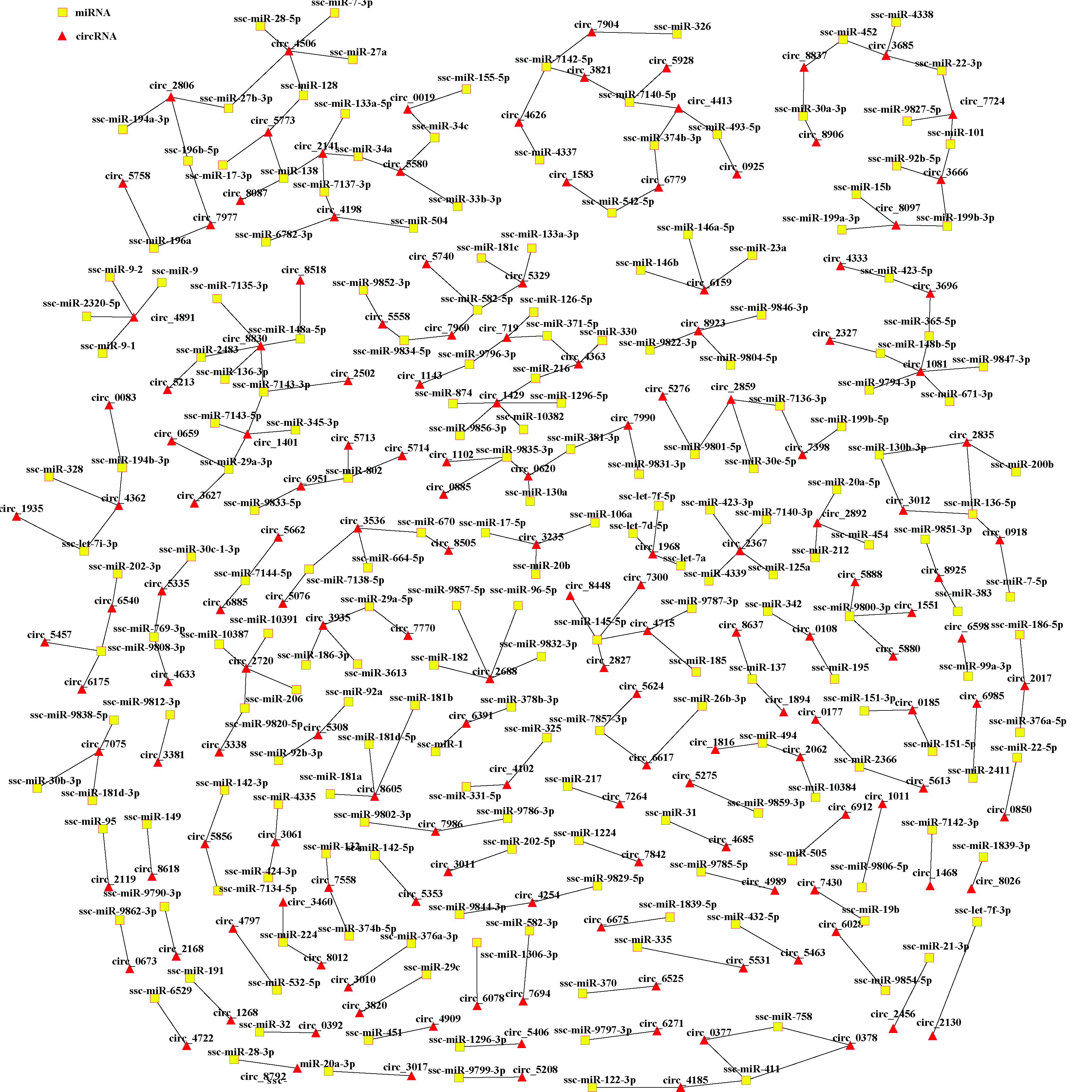

Supplement: Supplementary file 16 — Additional file 16. Fig. S1. Prediction of miRNA targets potentially sponged by DECs. [file 40813_2022_270_MOESM16_ESM.tif]

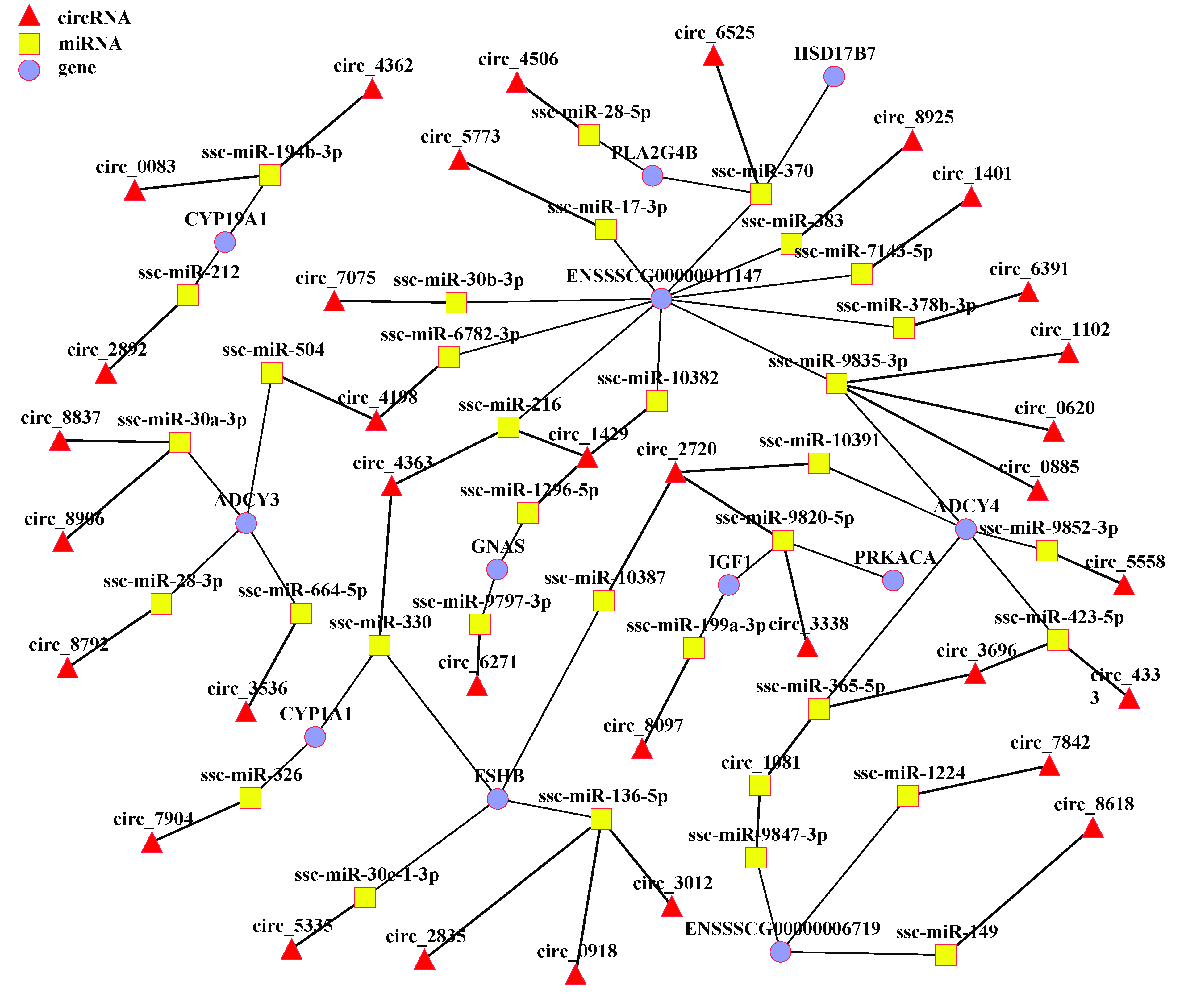

Supplement: Supplementary file 23 — Additional file 23. Fig. S8. The circRNA-miRNA-mRNA interactive networks of ovarian steroidogenesis pathway. [file 40813_2022_270_MOESM23_ESM.tiff]
